# Nearest shrunken centroids via alternative genewise shrinkages

**DOI:** 10.1371/journal.pone.0171068

**Published:** 2017-02-15

**Authors:** Byeong Yeob Choi, Eric Bair, Jae Won Lee

**Affiliations:** 1 Department of Epidemiology and Biostatistics, University of Texas Health Science Center, San Antonio, TX, United States of America; 2 Department of Biostatistics, University of North Carolina, Chapel Hill, NC, United States of America; 3 Department of Endodontics, University of North Carolina, Chapel Hill, NC, United States of America; 4 Department of Statistics, Korea University, Anam-Dong, Seoul, South Korea; Universitatsmedizin Greifswald, GERMANY

## Abstract

Nearest shrunken centroids (NSC) is a popular classification method for microarray data. NSC calculates centroids for each class and “shrinks” the centroids toward 0 using soft thresholding. Future observations are then assigned to the class with the minimum distance between the observation and the (shrunken) centroid. Under certain conditions the soft shrinkage used by NSC is equivalent to a LASSO penalty. However, this penalty can produce biased estimates when the true coefficients are large. In addition, NSC ignores the fact that multiple measures of the same gene are likely to be related to one another. We consider several alternative genewise shrinkage methods to address the aforementioned shortcomings of NSC. Three alternative penalties were considered: the smoothly clipped absolute deviation (SCAD), the adaptive LASSO (ADA), and the minimax concave penalty (MCP). We also showed that NSC can be performed in a genewise manner. Classification methods were derived for each alternative shrinkage method or alternative genewise penalty, and the performance of each new classification method was compared with that of conventional NSC on several simulated and real microarray data sets. Moreover, we applied the geometric mean approach for the alternative penalty functions. In general the alternative (genewise) penalties required fewer genes than NSC. The geometric mean of the class-specific prediction accuracies was improved, as well as the overall predictive accuracy in some cases. These results indicate that these alternative penalties should be considered when using NSC.

## Introduction

Nearest shrunken centroids (NSC) is one of the most frequently used classification methods for high-dimensional data such as microarray data [1, 2]. NSC shrinks the average expression (i.e., centroid) of each gene within each class toward the overall centroid via soft thresholding. Genes whose expression levels do not significantly differ between the classes will have their centroids reduced to the overall centroids, effectively removing them from the classification procedure. The amount of shrinkage is determined by cross validation. Then class prediction is performed using the shrunken centroids, which allows one to identify important genes and predict the class of unlabeled observations.

Wang and Zhu [3] showed that NSC is the solution to the regression problem that estimates the class centroids subject to an *L*_1_ penalty (i.e., LASSO) of Tibshirani [4]. They observed that the LASSO penalty applies the same penalties to all centroids, but the centroids for the same gene should be treated as one group. To overcome this problem, they proposed two NSC methods using different penalties: adaptive *L*_∞_-norm penalized NSC (ALP-NSC) and adaptive hierarchically penalized NSC (AHP-NSC). They showed that the two NSC methods have better performance than the original NSC in terms of misclassification error rate and the number of variables with nonzero centroids. However, ALP-NSC requires an exhaustive search to find an index set satisfying certain condition. If no such indices exist, quadratic programming must be employed to estimate the parameters. AHP-NSC requires an iterative procedure to estimate the parameters, and this increases the computational burden as the number of genes increases.

While Wang and Zhu [3] sought to improve NSC by considering the correlation between the centroids for the same gene, Guo et al. [5] improved NSC by regularizing the covariance matrix of genes in addition to shrinking the class centroids. In fact, Guo et al. [5] modified the classical linear discriminant score, not the diagonal linear discriminant score, and thus the method of Guo et al. [5] is a generalized version of NSC. Pang et al. [6] proposed an improved diagonal linear discriminant analysis (LDA) through shrinkage and regularization of the variances, but their method dose not perform variable selection. Several authors proposed new types of sparse LDA and provided the related optimality conditions and asymptotic properties. Shao et al. [7] applied the thresholding methodology, which was developed for function estimation, to the estimation of the means and variances, and Mai et al. [8] used the least squares formulation of LDA.

Another way to improve NSC is to modify the way to select an optimal threshold as in Blagus and Lusa [9]. They improved NSC in class-imbalanced data by selecting the optimal threshold as the value that maximizes the geometric mean of the class-specific prediction accuracies. Their numerical studies showed that the modified NSC improved the prediction accuracy of the minority class and area under the curve (AUC), and even the average prediction accuracy of entire classes for some real data.

In this article, we proposed the methods that improve NSC through alternative shrinkage of the class centroids. Like Wang and Zhu [3], we used an additional parameter, which controls the amount of penalization given to the parameters for our methods. These alternative shrinkages were derived from three existing alternative penalized regression methods, namely the smoothly clipped absolute deviation (SCAD) [10], the adaptive LASSO (ADA) [11], and the minimax concave penalty (MCP) [12], which are known to outperform LASSO regression in some situations. They enjoy the oracle property, which means that the efficiency of these estimators is not reduced when the subset of variables with nonzero coefficients is unknown. As noted earlier, under an orthonormal design (such as the case of NSC), the LASSO solution can be obtained via soft thresholding. Similarly, these three regression methods also have simple solutions in the NSC setting, so the computation is easy and fast. While the LASSO solution yields biased estimates for large coefficients, these methods produce unbiased estimates. Several researchers have considered the use of the alternative shrinkage methods in place of soft shrinkage [2, 13, 14]. In this article, we will evaluate the performances of these alternative shrinkage methods by comparing them with conventional soft shrinkage systematically through simulation and real data studies.

Blockwise additive penalties, which were discussed in Antoniadis and Fan [15], were shown to give alternative genewise shrinkage estimators of the class centroids in the NSC setting, where the block is the gene. Similar to the methods of Wang and Zhu [3], these estimators use the fact that the centroids from the same gene should be treated as a group, but they are less computationally intensive than those of Wang and Zhu [3] because an iterative procedure is not involved. The approach of Blagus and Lusa [9] was also applied for our alternative (genewise) penalties to further improve NSC, especially for class-imbalanced data.

In the Methods section, we described the penalized least squares framework for general shrinkage methods using the model of Wang and Zhu [3], which includes the special case of NSC. We examined the performance of NSC with alternative penalty functions (ALT-NSC), which include the SCAD, the adaptive LASSO and the MCP. We also described how ALT-NSC can be used for genewise inference (GEN-NSC). In the Simulation section, we conducted simulation studies and showed that ALT-NSC and GEN-NSC have substantially better performance than NSC in terms of predictive accuracy and feature selection in data sets with multiple classes. In the Real Data Study section, we applied the proposed variants of NSC to several real microarray data sets. A discussion and concluding remarks are provided in the last two sections.

## Methods

### Penalized least squares for the nearest shrunken centroids

Adapting the framework of Wang and Zhu [3], let *x*_*ij*_ be the gene expression for the *j*th gene of the *i*th sample (*j* = 1, …, *p*; *i* = 1, …, *n*). There are *K* classes and each sample *i* belongs to one of *K* classes, that is *i* ∈ *C*_*k*_, where *C*_*k*_ is the set of sample indexes belonging to class *k* ∈ {1, …, *K*}, and *n*_*k*_ is the number of samples for class *k*. The average expressions for the *j*th gene in the *k*th class and over the entire data set are ${\bar{x}}_{kj}=\sum_{i\in C_{k}}x_{ij}/n_{k}$ and ${\bar{x}}_{j}=\sum_{i=1}^{n}x_{ij}/n$ respectively.

Let

$$\begin{array}{c} \mu_{kj}^{0}=\frac{{\bar{x}}_{kj}-{\bar{x}}_{j}}{m_{k}s_{j}}, \end{array}$$

where *s*_*j*_ is the pooled within-class standard deviation for the *j*th gene:

$$\begin{array}{c} s_{j}^{2}=\frac{1}{n-K}\sum_{k=1}^{K}\sum_{i\in C_{k}}{\left(x_{ij}-{\bar{x}}_{kj}\right)}^{2}, \end{array}$$

and $m_{k}=\sqrt{1/n_{k}-1/n}$. Alternatively, *s*_*j*_ + *s*_0_ can be used instead of *s*_*j*_ to prevent the genes with low expression levels from having large $\mu_{kj}^{0}$ values by chance due to very small *s*_*j*_ values, where *s*_0_ is a small constant. The statistic $\mu_{kj}^{0}$ is equivalent to *d*_*kj*_, in Tibshirani *et al*. [1, 2], which is a t-statistic for the *j*th gene comparing class *k* to the average of the other classes.

Let $y_{ij}=\left(x_{ij}-{\bar{x}}_{j}\right)/\left(m_{k}s_{j}\right)$ and consider the following linear model:

$$\begin{array}{c} y_{ij}=\sum_{k=1}^{K}z_{ik}\mu_{kj}+\varepsilon_{ij}, \end{array}$$ (1)

where *z*_*ik*_ = 1 if sample *i* belongs to the class *k*, and 0 otherwise, *μ*_*kj*_ is a parameter to be estimated and *ε*_*ij*_ is an independent error term that has variance $1/m_{k}^{2}$ if sample *i* belongs to class *k*. For a fixed gene index *j*, *μ*_*kj*_ is a deviation from the overall mean, so we have the constraint that $\sum_{k=1}^{K}\mu_{kj}=0$. The class index to which sample *i* belongs is denoted by *k*(*i*) ∈ {1, …, *K*}. By multiplying $1/\sqrt{n_{k(i)}}$ to both sides in Eq (1), we have

$$\begin{array}{c} y_{ij}^{*}=\sum_{k=1}^{K}\frac{z_{ik}}{\sqrt{n_{k(i)}}}\mu_{kj}+\varepsilon_{ij}^{*}, \end{array}$$ (2)

where $y_{ij}^{*}=y_{ij}/\sqrt{n_{k(i)}}$ and $\varepsilon_{ij}^{*}=\varepsilon_{ij}/\sqrt{n_{k(i)}}$. In vector notation, Eq (2) can be written as

$$\begin{array}{c} y^{*}=W\mu +\varepsilon^{*}, \end{array}$$

where

$$\begin{array}{ccc} y^{*} & = & {\left(\frac{y_{11}}{\sqrt{n_{k(1)}}},\frac{y_{12}}{\sqrt{n_{k(1)}}},...,\frac{y_{1p}}{\sqrt{n_{k(1)}}},\frac{y_{21}}{\sqrt{n_{k(2)}}},...,\frac{y_{np}}{\sqrt{n_{k(n)}}}\right)}^{T},\\ \varepsilon^{*} & = & {\left(\frac{\varepsilon_{11}}{\sqrt{n_{k(1)}}},\frac{\varepsilon_{12}}{\sqrt{n_{k(1)}}},...,\frac{\varepsilon_{1p}}{\sqrt{n_{k(1)}}},\frac{\varepsilon_{21}}{\sqrt{n_{k(2)}}},...,\frac{\varepsilon_{np}}{\sqrt{n_{k(n)}}}\right)}^{T},\\ \mu & = & {\left(\mu_{11},\mu_{12},...,\mu_{1p},\mu_{21},...,\mu_{Kp}\right)}^{T},\\ \mu^{0} & = & {\left(\mu_{11}^{0},\mu_{12}^{0},...,\mu_{1p}^{0},\mu_{21}^{0},...,\mu_{Kp}^{0}\right)}^{T}, \end{array}$$

where *A*^*T*^ denotes the transpose of a vector or matrix *A*. The design matrix **W** = (**W**_1_, …, **W**_*Kp*_) is an *np* × *Kp* matrix, where **W**_*l*_ is a *np* × 1 vector that corresponds to the *l*th element of the vector *μ* for *l* = 1, …, *Kp*. If an index *l* belongs to a class index *k* and a gene index *j*, then *n*_*k*(*i*)_ elements of **W**_*l*_ are $1/\sqrt{n_{k(i)}}$ and the rest of the elements are zeros because there are exactly *n*_*k*(*i*)_ samples belonging to class *k*(*i*). This implies that $W_{l}^{T}W_{l}=1\;\left(l=1,...,Kp\right)$. In addition, we can see that each row of **W** has only one non-zero value and the rest of the elements are zero. This is because each *y*_*ij*_ takes only one *μ*_*kj*_, and this implies $W_{l}^{T}W_{h}=0$ for (*l* ≠ *h*). Thus, **W** is orthonormal. Note that ***μ***^0^ = **W**^*T*^ y* is the least squares estimator for **μ**, and let ${\hat{y}}^{*}=W\mu^{0}$. Since **W** is orthonormal, a form of the penalized least squares is given by [10]:

$$\begin{array}{l} \frac{1}{2}∥y^{*}-W\mu {∥}^{2}+\lambda \sum_{j=1}^{p}\sum_{k=1}^{K}p(\left|\mu_{kj}\right|)\\ =\frac{1}{2}∥y^{*}-{\hat{y}}^{*}{∥}^{2}+\frac{1}{2}\sum_{j=1}^{p}\sum_{k=1}^{K}{\left(\mu_{kj}^{0}-\mu_{kj}\right)}^{2}+\sum_{j=1}^{p}\sum_{k=1}^{K}p_{\lambda }(\left|\mu_{kj}\right|), \end{array}$$ (3)

where *p*_*λ*_(⋅) = *λp*(⋅) is a penalty function and ${∥A∥}^{2}=\sum_{i=}^{n}a_{i}^{2}$ when *A* = (*a*_1_, …, *a*_*n*_)^*T*^. The problem of minimizing Eq (3) with respect to *μ*_*kj*_ is equivalent to minimizing it componentwise. By ignoring $\frac{1}{2}{∥y^{*}-{\hat{y}}^{*}∥}^{2}$, which is irrelevant to the parameters, this allows us to consider the following penalized least squares problem:

$$\begin{array}{c} \frac{1}{2}{\left(\mu_{kj}^{0}-\mu_{kj}\right)}^{2}+p_{\lambda }\left(\right|\mu_{kj}\left|\right). \end{array}$$ (4)

Eq (4) shows that the minimization problem Eq (3) has been converted to a univariate minimization problem. Since, the univariate solutions for regression coefficients are presented in the papers describing these penalized regression methods, we can use these solutions to obtain ${\hat{\mu }}_{kj}$. NSC uses the LASSO penalty function *p*_*λ*_(|*μ*_*kj*_|) = *λ*|*μ*_*kj*_| [4], and the resulting estimator for *μ*_*kj*_ is given by

$$\begin{array}{c} {\hat{\mu }}_{kj}=\text{sgn}\left(\mu_{kj}^{0}\right)\left(\right|\mu_{kj}^{0}{\left|-\lambda \right)}_{+}, \end{array}$$

where “sgn” is a sign function and *z*_+_ is the positive part of *z*. The LASSO solution is equivalent to the soft shrinkage estimate [16]. The resulting estimators for *μ*_*kj*_ under alternative penalties are presented in the next subsection.

To predict the class of a new sample $x^{*}={\left(x_{1}^{*},...,x_{p}^{*}\right)}^{T}$, we define the discriminant score for class *k* as

$$\begin{array}{c} \delta_{k}\left(x^{*}\right)=\sum_{j=1}^{p}\frac{{\left(x_{j}^{*}-{\hat{x}}_{kj}\right)}^{2}}{s_{j}^{2}}-2\log\pi_{k}, \end{array}$$

where ${\hat{x}}_{kj}={\bar{x}}_{j}+{\hat{\mu }}_{kj}m_{k}s_{j}$ is a shrunken mean and *π*_*k*_ = *n*_*k*_/*n* is a prior probability estimate for class *k*. The shrunken mean and the discriminant score depend on the shrinkage method used, hence the choice of shrinkage method affects class prediction and gene selection. Finally, the classification rule is given by

$$\begin{array}{c} C\left(x^{*}\right)=k^{*},\;\text{where}\;k^{*}=\arg\min_{k}\delta_{k}\left(x^{*}\right). \end{array}$$

### Alternative shrinkage methods (ALT-NSC)

Here, we described several shrinkage methods that are possible alternatives to soft shrinkage. The first order derivative of the SCAD penalty function [10] is defined as

$$\begin{array}{c} p_{\lambda ,a}^{\prime }\left(\right|\mu_{kj}\left|\right)=\lambda \left\{I(|\mu_{kj}|\leq \lambda )+\frac{{\left(a\lambda -\mu_{kj}\right)}_{+}}{(a-1)\lambda }I(|\mu_{kj}\left|>\lambda \right)\right\}, \end{array}$$

for some *a* > 2. This penalty function gives smaller penalties on larger coefficients. The resulting estimator for *μ*_*kj*_ is

$$\begin{array}{c} {\hat{\mu }}_{kj}=\left\{\begin{array}{cc} \text{sgn}\left(\mu_{kj}^{0}\right)\left(\right|\mu_{kj}^{0}{\left|-\lambda \right)}_{+}, & \text{if}\;|\mu_{kj}^{0}|\leq 2\lambda \\ \frac{\left(a-1\right)\mu_{kj}^{0}-\text{sgn}\left(\mu_{kj}^{0}\right)a\lambda }{a-2}, & \text{if}\;2\lambda <|\mu_{kj}^{0}|\leq a\lambda \\ \mu_{kj}^{0}, & \text{if}\;|\mu_{kj}^{0}|>a\lambda . \end{array}\right. \end{array}$$

If *a* is close to 2, then SCAD behaves like a hard shrinkage estimate when estimating *μ*_*kj*_.

The adaptive LASSO penalty function [11], which is the LASSO penalty function with a data-dependent weight, is given by

$$\begin{array}{c} p_{\lambda ,a}\left(\right|\mu_{kj}\left|)=\lambda \right|\mu_{kj}\left|/\right|\mu_{kj}^{0}{|}^{a}, \end{array}$$

where *a* > 0. The resulting solution is

$$\begin{array}{c} {\hat{\mu }}_{kj}=\text{sgn}\left(\mu_{kj}^{0}\right)\left(\right|\mu_{kj}^{0}\left|-\lambda /\right|\mu_{kj}^{0}{{|}^{a})}_{+}, \end{array}$$

or

$$\begin{array}{c} {\hat{\mu }}_{kj}=\mu_{kj}^{0}\left(1-\lambda /\right|\mu_{kj}^{0}{{|}^{a+1})}_{+}. \end{array}$$

The adaptive LASSO solution is equivalent to soft shrinkage when *a* = 0 and is similar to the nonnegative garotte when *a* = 1 [17] (although the nonnegative garotte requires additional sign restrictions).

The MCP penalty function [12] is defined as

$$\begin{array}{c} p_{\lambda ,a}\left(\right|\mu_{kj}\left|\right)=\left\{\begin{array}{cc} \lambda |\mu_{kj}|-\mu_{kj}^{2}/\left(2a\right), & \text{if}\;|\mu_{kj}|\leq a\lambda ,\\ 0.5a\lambda^{2}, & \text{if}\;|\mu_{kj}|>a\lambda , \end{array}\right. \end{array}$$

where *a* > 1. The resulting solution is given by

$$\begin{array}{c} {\hat{\mu }}_{kj}=\left\{\begin{array}{cc} \frac{\text{sgn}\left(\mu_{kj}^{0}\right)\left(\right|\mu_{kj}^{0}{\left|-\lambda \right)}_{+}}{1-1/a}, & \text{if}\;|\mu_{kj}^{0}|\leq a\lambda ,\\ \mu_{kj}^{0}, & \text{if}\;|\mu_{kj}^{0}|>a\lambda . \end{array}\right. \end{array}$$

The MCP solution is equivalent to firm shrinkage, which offers advantages over soft and hard shrinkage [18]. The MCP solution approaches hard shrinkage as *a* → 1 and soft shrinkage as *a* → ∞.

As mentioned previously, these shrinkage methods are known to have oracle properties under some mild conditions (for details, see [10], [11] and [12]). The LASSO solution is inconsistent because it produces estimates biased toward zero. This bias in the LASSO can also cause its variable selection to be inconsistent [12]. The basic reason that the alternative shrinkages can produce better estimates is because they have different rules for estimating the coefficients *μ*_*kj*_, which depend on the size of |*μ*_*kj*_|. When the sizes of the coefficients are large, these procedures leave them almost unpenalized (or completely unpenalized). Thus, they overcome the tendency of soft shrinkage to produce biased estimates.

While soft shrinkage has one tuning parameter *λ*, the alternative shrinkage methods have two tuning parameters, namely *a* and *λ*. The tuning parameter *a* controls the size of the penalties for large coefficients. The tuning parameters are determined by cross validation (CV). In our subsequent analysis, six values of the tuning parameter *a* were examined for each ALT-NSC and genewise shrinkage method: (0.5, 1, 1.5, 2, 2.5, 3) for the adaptive LASSO penalty, (2.01, 2.2, 2.5, 2.8, 3.2, 3.7) for the SCAD penalty and (1.01, 1.3, 1.7, 2, 2.5, 3) for the MCP penalty. For each method, thirty values of *λ* were considered. For the case when there are ties among the CV prediction accuracies or g-means, we chose the parameters resulting in a smaller number of genes.

### Genewise shrinkage methods (GEN-NSC)

Here we extend the shrinkage methods discussed in the previous subsection to genewise inference. Let ***μ***_*j*_ = (*μ*_1*j*_, …, *μ*_*Kj*_)^*T*^ denote a *K* × 1 mean vector for the *j*th gene. Further let $\mu_{j}^{0}={\left(\mu_{1j}^{0},...,\mu_{Kj}^{0}\right)}^{T}$ denote the corresponding mean estimator vector. The objective function to be minimized for the genewise penalized least squares estimator is

$$\begin{array}{c} \frac{1}{2}∥y^{*}{-W\mu ∥}^{2}+\lambda \sum_{j=1}^{p}p(∥\mu_{j}∥). \end{array}$$ (5)

Note that instead of penalizing *μ*_*kj*_, we penalize the vector *μ*_*j*_. Using the fact that

$$\begin{array}{c} \sum_{j=1}^{p}\sum_{k=1}^{K}{\left(\mu_{kj}^{0}-\mu_{kj}\right)}^{2}=\sum_{j=1}^{p}{∥\mu_{j}^{0}-\mu_{j}∥}^{2} \end{array}$$

and the orthonormality of **W**, Eq (5) can be written as

$$\begin{array}{c} \frac{1}{2}∥y^{*}-{\hat{y}}^{*}{∥}^{2}+\frac{1}{2}\sum_{j=1}^{p}∥\mu_{j}^{0}-\mu_{j}{∥}^{2}+\sum_{j=1}^{p}p_{\lambda }(∥\mu_{j}∥). \end{array}$$ (6)

The solution to Eq (6) is genewise separable, and thus one may solve it by minimizing

$$\begin{array}{c} \frac{1}{2}∥\mu_{j}^{0}-\mu_{j}{∥}^{2}+p_{\lambda }(∥\mu_{j}∥). \end{array}$$ (7)

Using the result of Antoniadis *et al*. [15], the solution to Eq (7) is given by

$$\begin{array}{c} {\hat{\mu }}_{j}=r(∥\mu_{j}^{0}∥)\mu_{j}^{0}/∥\mu_{j}^{0}∥, \end{array}$$ (8)

where $r(∥\mu_{j}^{0}∥)$ is the solution to

$$\begin{array}{c} \min_{r}\{(∥\mu_{j}^{0}{∥-r)}^{2}+p_{\lambda }\left(r\right)\}. \end{array}$$ (9)

Since Eq (8) depends on the penalty function *p*_*λ*_(⋅), we can derive genewise shrinkage methods under diverse penalty functions. Note that the problem of solving Eq (9) is equivalent to that of Eq (4), and thus, the computational complexity of the genewise shrinkages is the same as that of the alternative shrinkages.

When the LASSO penalty is employed,

$$\begin{array}{c} {\hat{\mu }}_{j}=\mu_{j}^{0}(1-\lambda /∥\mu_{j}^{0}{∥)}_{+}. \end{array}$$

If the SCAD penalty is used,

$$\begin{array}{c} {\hat{\mu }}_{j}=\left\{\begin{array}{cc} \mu_{j}^{0}(1-\lambda /∥\mu_{j}^{0}{∥)}_{+}, & \text{if}\;∥\mu_{j}^{0}∥\leq 2\lambda ,\\ \frac{\left(a-1\right)∥\mu_{j}^{0}∥-a\lambda }{\left(a-2\right)∥\mu_{j}^{0}∥}\mu_{j}^{0}, & \text{if}\;2\lambda <∥\mu_{j}^{0}∥\leq a\lambda ,\\ \mu_{j}^{0}, & \text{if}\;∥\mu_{j}^{0}∥>a\lambda , \end{array}\right. \end{array}$$

where *a* > 2. For the adaptive LASSO penalty, the resulting solution is

$$\begin{array}{c} {\hat{\mu }}_{j}=\mu_{j}^{0}(1-\lambda /∥\mu_{j}^{0}{{∥}^{a+1})}_{+}. \end{array}$$

For the MCP penalty,

$$\begin{array}{c} {\hat{\mu }}_{j}=\left\{\begin{array}{cc} \frac{\mu_{j}^{0}(1-\lambda /∥\mu_{j}^{0}{∥)}_{+}}{1-1/a}, & \text{if}\;∥\mu_{j}^{0}∥\leq a\lambda ,\\ \mu_{j}^{0}, & \text{if}\;∥\mu_{j}^{0}∥>a\lambda , \end{array}\right. \end{array}$$

where *a* > 1.

The thresholding rules of the genewise shrinkage methods are determined by $∥\mu_{j}^{0}∥$ instead of an individual $\mu_{kj}^{0}$. By pulling information from the neighboring mean estimators belonging to the same gene, the genewise shrinkage may allow the accuracy of the thresholding mean estimators to be improved. Furthermore, Eq (5) has a nice Bayesian interpretation [15]: the genewise penalized least squares method models the mean coefficients belonging to the same gene by using proper prior distributions.

### Geometric mean methods (GM)

Adpating the idea of Blagus and Lusa [9], we considered the optimal values of tuning parameters (*a*, *λ*) of the (genewise) althernative shrinkage methods to be those that maximize the geometric mean of the class-specific prediction accuracies.

Throughout the remainder of this manuscript, we will refer to the genewise version of each method by adding “G” to the beginning of its abbreviated name. Moreover, when the tuning parameters are determined by the geometric mean, we will add “GM-” to the beginning of the name. For example, “GADA” refers to the genewise version of adaptive lasso, and “GM-GADA” referes to “GADA” whose tunnig parameters are determined by the geometric mean.

## Simulations

In this section, we conducted simulation studies to compare ALT-NSC, GEN-NSC, and the GM versions of ALT-NSC and GEN-NSC to conventional NSC. We examined the overall prediction accuracy (PA), geometric mean (g-mean), area under the curve (AUC, only for a two-class classification scenario), sensitivity (SEN) and positive predictive value (PPV). SEN is the number of detected important genes divided by total number of important genes. PPV is the number of detected important genes divided by total number of genes the method selects. As in Dudoit et al. [19], we presented the median and upper quartiles of the evaluation measures.

In a two class classification scenario, we generated two classes from multivariate normal distributions with sample sizes, *n*_1_ = *nπ*_1_ and *n* − *n*_1_: MVN(*μ*_1_, *Σ*) and MVN(*μ*_2_, *Σ*), each had a dimension of *p* = 2500. *μ*_1_ was equal to 0 for all genes and *μ*_2_ was 0.5 for 100 genes and 0 for the rest of genes. The differentially expressed (DE) 100 genes were randomly selected. As in Guo et al. [5] and Pang et al. [6], *Σ* was a block diagonal matrix with each diagonal block *Σ*_*ρ*_ having an auto-regressive structure and alternating in sign. The block size was 50 × 50 and there were 50 blocks, which gave a total of 2500 genes:

$$\begin{array}{c} \Sigma_{\rho }=\left(\begin{array}{ccccc} 1 & \rho & \cdots & \rho^{48} & \rho^{49}\\ \rho & 1 & \ddots & \cdots & \rho^{48}\\ \vdots & \ddots & \ddots & \ddots & \vdots \\ \rho^{48} & \cdots & \ddots & 1 & \rho \\ \rho^{49} & \rho^{48} & \cdots & \rho & 1 \end{array}\right). \end{array}$$

*ρ* took values of 0.5 and 0.9, indicating sparse and dense correlation blocks, and *π*_1_ took values of 0.5 and 0.8, corresponding to class-balance and -imbalance.

The three class classification scenario is very similar to the previous one. We generated three classes from multivariate normal distributions with the fixed proportions (*π*_1_, *π*_2_, *π*_3_) = (0.4, 0.2, 0.4): MVN(*μ*_1_, *Σ*), MVN(*μ*_2_, *Σ*) and MVN(*μ*_3_, *Σ*), each of which had the same dimension as the previous scenario. Ninety differentially expressed genes were randomly selected and those DE genes had mean vectors of (*γ*, 0,−*γ*). We used the same *Σ* as in the first simulation. We let *γ* take the values of 0.5 and 0.1 to study how the effect size of DE genes is related to the performances of the classifiers.

Given *a*, the tuning parameter *λ* was chosen to minimize the *m*-fold CV misclassification error rate on training data set, and we let *m* = 5. We generated training data sets with sample size 100 and test data sets with 10 times the sample size of the training data. Then test error rates were computed using the tuning parameters selected by CV. Gene selection was performed in the same way as in Tibshirani *et al*. [1, 2], where the genes with at least one nonzero difference were selected (the *j*th gene is selected if there exists at least one *k* such that ${\hat{\mu }}_{kj}\neq 0$).

Simulation results for the two-class scenario have been presented in Tables 1, 2, 3 and 4. All of the proposed methods performed very similarly to NSC in terms of PA, g-mean and AUC except when the diagonal block matrix was dense and class was imbalanced; ALT-NSC improved the g-mean slightly, the GM versions of ALT-NSC and GEN-NSC also improved the g-mean, and GM-GSCAD had the highest g-mean in this setting. The classifiers had poorer prediction perfromance based on PA, g-mean and AUC when the block diagonal matrix was dense and classes were imbalanced. Gene selection accuracy (SEN and PPV) also decreased when class was imbalanced.

**Table 1:** Two groups with sparse block diagonal structure (*ρ* = 0.5) and class-balance (*π*_1_ = 0.5).

Method	PA		g-mean		AUC		SEN		PPV	
	Median	Upper	Median	Upper	Median	Upper	Median	Upper	Median	Upper
NSC	91.9	93.2	91.9	93.2	97.8	98.3	69.5	78.0	37.6	52.2
ADA	91.7	93.2	91.7	93.2	97.8	98.3	65.5	77.0	41.5	55.2
SCAD	92.1	92.8	92.1	92.8	97.8	98.1	81.0	87.0	24.3	33.1
MCP	91.7	92.7	91.6	92.7	97.6	98.1	58.5	70.2	53.1	64.4
GM-NSC	91.9	93.2	91.9	93.2	97.9	98.2	70.0	78.2	37.2	51.0
GM-ADA	91.9	93.2	91.8	93.2	97.9	98.3	67.0	77.2	39.2	53.2
GM-SCAD	92.1	93.0	92.1	93.0	97.8	98.1	81.0	87.2	24.3	32.4
GM-MCP	91.6	92.8	91.6	92.8	97.6	98.1	58.5	71.2	52.6	64.1
GNSC	91.9	93.2	91.9	93.2	97.7	98.3	66.0	75.2	42.4	51.9
GADA	92.0	93.3	92.0	93.3	97.9	98.3	66.0	75.2	43.0	56.5
GSCAD	91.7	92.9	91.7	92.9	97.7	98.2	79.0	85.0	27.0	36.2
GMCP	91.8	93.0	91.8	93.0	97.7	98.2	60.5	71.2	52.1	62.0
GM-GNSC	92.0	93.3	92.0	93.3	97.8	98.3	67.0	76.5	41.1	51.3
GM-GADA	92.0	93.3	92.0	93.3	97.9	98.3	66.0	75.2	43.1	56.5
GM-GSCAD	92.0	92.9	91.9	92.9	97.8	98.2	80.0	86.0	24.9	36.3
GM-GMCP	91.9	93.0	91.8	92.9	97.7	98.2	61.0	72.2	51.3	61.4

“PA”, “g-mean” and “AUC” are overall accuracy, geometric mean and AUC of class prediction, calculated from the test data set. “SEN” and “PPV” are sensitivity and positive predictive value of gene selection obtained from the training data set. “Median” and “Upper” are median and upper quartiles of 100 repetitions. The scale of all the numbers is a percentage.

**Table 2:** Two groups with dense block diagonal structure (*ρ* = 0.9) and class-balance (*π*_1_ = 0.5).

Method	PA		g-mean		AUC		SEN		PPV	
	Median	Upper	Median	Upper	Median	Upper	Median	Upper	Median	Upper
NSC	85.8	88.1	85.7	88.1	93.4	95.4	46.0	57.0	68.4	81.4
ADA	86.1	88.7	86.1	88.7	93.4	95.5	44.0	57.0	71.9	81.4
SCAD	85.3	87.8	85.3	87.8	93.1	95.1	51.5	65.0	61.5	78.3
MCP	86.0	88.6	86.0	88.6	93.5	95.6	34.5	47.0	81.8	90.7
GM-NSC	85.9	88.4	85.9	88.4	93.6	95.4	48.5	58.5	64.6	79.0
GM-ADA	86.1	88.6	86.1	88.5	93.4	95.5	45.0	59.0	69.0	80.1
GM-SCAD	85.3	87.9	85.3	87.9	93.3	95.1	54.0	66.2	60.2	72.7
GM-MCP	86.3	88.6	86.3	88.6	93.7	95.6	36.0	48.0	81.4	91.2
GNSC	85.0	87.9	85.0	87.9	93.1	95.2	47.0	54.0	69.9	81.9
GADA	84.8	88.0	84.8	88.0	92.9	95.3	45.0	55.0	73.0	83.8
GSCAD	84.3	87.1	84.3	87.1	92.9	94.5	50.0	64.2	63.5	80.9
GMCP	84.2	88.2	84.2	88.2	92.4	95.5	34.5	47.2	81.2	94.9
GM-GNSC	85.0	87.8	85.0	87.8	93.1	95.2	48.0	56.5	66.7	81.9
GM-GADA	84.7	88.3	84.7	88.3	92.8	95.3	44.5	55.0	72.3	83.8
GM-GSCAD	84.3	87.4	84.3	87.4	92.9	94.6	51.5	66.5	60.3	80.1
GM-GMCP	84.5	88.3	84.5	88.3	92.7	95.6	36.0	50.0	80.9	92.7

**Table 3:** Two groups with sparse block diagonal structure (*ρ* = 0.5) and class-imbalance (*π*_1_ = 0.8).

Method	PA		g-mean		AUC		SEN		PPV	
	Median	Upper	Median	Upper	Median	Upper	Median	Upper	Median	Upper
NSC	84.5	85.3	48.4	52.8	93.4	94.4	66.0	74.0	18.4	23.7
ADA	84.5	85.3	47.9	52.8	93.2	94.3	58.0	64.0	23.1	33.8
SCAD	84.7	85.3	49.0	52.4	93.5	94.4	65.0	71.2	19.6	22.8
MCP	83.8	84.5	43.6	49.4	92.1	94.0	41.5	56.0	38.1	55.1
GM-NSC	84.5	85.4	48.5	52.8	93.4	94.4	66.5	75.2	18.1	21.7
GM-ADA	84.5	85.3	48.1	52.8	93.2	94.3	58.0	64.0	23.1	32.2
GM-SCAD	84.7	85.3	49.0	52.4	93.5	94.4	65.5	72.0	19.4	22.7
GM-MCP	83.9	84.8	44.7	49.9	92.3	94.1	44.0	56.2	37.4	50.7
GNSC	84.4	85.4	48.2	52.8	93.3	94.5	65.5	73.2	19.0	24.6
GADA	84.4	85.3	47.9	52.1	93.5	94.5	56.0	67.0	26.3	32.1
GSCAD	84.3	85.3	47.6	52.0	93.4	94.4	64.0	69.0	20.6	24.0
GMCP	83.8	84.9	44.9	50.0	92.1	94.0	44.5	58.0	34.3	54.0
GM-GNSC	84.5	85.4	48.4	52.8	93.3	94.5	66.0	74.0	18.8	23.9
GM-GADA	84.4	85.3	47.9	52.1	93.5	94.5	56.5	67.0	26.3	31.5
GM-GSCAD	84.5	85.3	49.0	52.8	93.5	94.4	64.0	69.5	19.8	23.4
GM-GMCP	83.9	84.9	45.2	50.0	92.2	94.1	46.0	58.2	32.5	53.2

**Table 4:** Two groups with dense block diagonal structure (*ρ* = 0.9) and class-imbalance (*π*_1_ = 0.8).

Method	PA		g-mean		AUC		SEN		PPV	
	Median	Upper	Median	Upper	Median	Upper	Median	Upper	Median	Upper
NSC	82.4	83.6	47.2	53.2	82.6	86.7	54.0	64.2	29.6	38.0
ADA	83.1	84.2	50.7	56.2	83.9	88.2	49.0	60.0	35.3	46.1
SCAD	82.9	84.4	52.3	57.2	83.5	87.0	59.0	70.0	26.3	34.2
MCP	83.1	84.2	49.4	53.8	83.4	87.6	36.0	52.2	42.2	67.0
GM-NSC	82.5	83.7	56.3	59.4	80.8	84.3	66.0	72.0	20.0	24.8
GM-ADA	83.1	84.4	55.9	59.6	82.7	86.0	58.0	64.2	26.6	33.9
GM-SCAD	83.2	84.5	56.6	60.0	82.2	85.7	66.0	74.0	19.6	25.0
GM-MCP	83.2	84.6	53.9	58.9	83.2	86.8	53.0	63.3	31.0	44.0
GNSC	82.5	83.4	45.1	53.4	81.7	86.4	51.0	62.2	30.1	39.9
GADA	82.8	84.1	50.2	56.3	82.8	87.8	44.0	57.2	35.8	48.1
GSCAD	82.7	84.0	49.5	55.3	83.0	86.3	53.5	71.0	27.7	37.4
GMCP	82.6	84.2	48.8	53.3	82.3	87.9	33.5	48.2	49.7	63.4
GM-GNSC	82.4	83.6	56.0	60.2	80.6	83.8	68.0	75.0	19.2	23.3
GM-GADA	82.8	84.2	55.1	59.7	81.6	86.1	57.0	69.5	26.4	35.4
GM-GSCAD	83.2	84.6	56.7	60.1	82.5	85.9	66.0	76.0	19.8	22.9
GM-GMCP	83.1	84.3	52.4	58.0	82.3	86.5	49.0	63.3	31.9	49.6

Simulation results for the three-class scenario have been presented in Tables 5, 6, 7 and 8. Unlike the two-class scenario, class sizes were always imbalanced. However, we considered different values of *γ*, which was the effect size of DE genes, and observed that our proposed methods performed significantly better than NSC. When the effect size of DE genes was moderate (*γ* = 0.5), only the ALT-NSC had better PA and g-mean, but GEN-NSC and GM methods showed no improvement. Under the very small effect size, all the classifiers performed very similarly in terms of PA, but their performance varied with respect to g-mean. MCP had the highest g-mean, and the other penalty functions gave zero as the median quartile of g-mean. Secondly, GM significantly improved g-mean for all the penalty functions, and the amount of the improvement was greater when the genewise penalties were used for the sparse block matrix. Finally, gene selection was also improved by GM: SEN increased and PPV stayed at almost the same value, compared to the corresponding methods based on the cross-validation prediction accuracy criterion.

**Table 5:** Three groups with class-imbalance (*π*_1_ = 0.4, *π*_2_ = 0.2, *π*_3_ = 0.4), sparse block diagonal structure (*ρ* = 0.5) and moderate mean difference (*γ* = 0.5).

Method	PA		g-mean		SEN		PPV	
	Median	Upper	Median	Upper	Median	Upper	Median	Upper
NSC	85.8	86.5	66.4	69.1	100.0	100.0	16.5	21.5
ADA	94.2	95.1	89.5	91.5	96.7	98.9	65.4	75.1
SCAD	95.5	96.3	92.4	93.9	100.0	100.0	24.2	28.9
MCP	96.4	97.2	94.6	95.7	85.0	90.3	94.2	97.4
GM-NSC	85.9	86.6	66.9	69.7	100.0	100.0	14.3	20.7
GM-ADA	94.2	95.3	89.6	91.6	96.7	98.9	66.0	75.3
GM-SCAD	95.6	96.3	92.6	94.3	100.0	100.0	23.5	28.5
GM-MCP	96.4	97.2	94.6	95.9	83.9	90.0	94.3	98.4
GNSC	85.3	86.2	64.8	68.3	100.0	100.0	20.4	29.0
GADA	92.2	93.4	85.1	87.6	96.7	97.8	71.7	81.6
GSCAD	93.5	94.5	88.4	90.4	100.0	100.0	31.2	40.7
GMCP	94.9	95.8	91.4	93.1	81.1	87.8	97.4	100.0
GM-GNSC	85.4	86.2	65.3	68.4	100.0	100.0	19.8	26.6
GM-GADA	92.2	93.4	85.4	87.7	96.1	97.8	72.4	82.9
GM-GSCAD	93.8	94.5	88.8	90.6	100.0	100.0	29.3	36.9
GM-GMCP	94.9	95.8	91.4	93.1	81.1	87.8	97.3	100.0

**Table 6:** Three groups with class-imbalance (*π*_1_ = 0.4, *π*_2_ = 0.2, *π*_3_ = 0.4), dense block diagonal structure (*ρ* = 0.9) and moderate mean difference (*γ* = 0.5).

Method	PA		g-mean		SEN		PPV	
	Median	Upper	Median	Upper	Median	Upper	Median	Upper
NSC	84.6	85.5	62.6	67.5	98.9	100.0	28.7	36.7
ADA	92.5	93.6	86.3	88.5	94.4	96.7	74.6	84.1
SCAD	92.0	93.0	86.5	88.3	98.9	100.0	33.2	41.5
MCP	95.4	96.3	93.5	94.8	81.7	87.8	97.4	100.0
GM-NSC	84.8	85.6	68.2	70.1	100.0	100.0	16.5	21.4
GM-ADA	92.7	93.7	86.5	88.7	95.6	96.7	73.6	80.8
GM-SCAD	92.0	93.4	87.2	89.5	99.4	100.0	27.8	33.0
GM-MCP	95.3	96.3	93.5	94.8	81.7	86.9	97.4	100.0
GNSC	84.3	85.1	62.0	66.8	98.9	100.0	37.8	49.4
GADA	90.8	91.7	82.0	84.6	94.4	96.7	82.4	90.9
GSCAD	90.8	91.8	83.7	85.9	98.9	100.0	38.4	47.7
GMCP	94.1	94.9	90.3	91.9	78.3	86.7	98.6	100.0
GM-GNSC	84.2	85.1	66.0	68.1	100.0	100.0	20.6	30.2
GM-GADA	90.9	91.9	82.9	85.1	95.6	97.8	78.3	86.8
GM-GSCAD	90.9	91.9	84.0	86.3	98.9	100.0	34.0	43.0
GM-GMCP	94.2	94.9	90.3	91.6	78.3	86.9	98.6	100.0

**Table 7:** Three groups with class-imbalance (*π*_1_ = 0.4, *π*_2_ = 0.2, *π*_3_ = 0.4), sparse block diagonal structure (*ρ* = 0.5) and small mean difference (*γ* = 0.1).

Method	PA		g-mean		SEN		PPV	
	Median	Upper	Median	Upper	Median	Upper	Median	Upper
NSC	42.8	44.3	0.0	18.0	12.2	65.6	5.4	11.4
ADA	42.8	44.0	0.0	20.1	12.2	58.1	5.3	10.2
SCAD	42.9	44.3	0.0	18.0	21.7	77.8	4.5	9.2
MCP	42.9	44.1	16.3	20.8	11.1	53.9	5.9	11.2
GM-NSC	42.4	43.8	19.5	24.4	43.3	72.5	4.4	5.7
GM-ADA	42.8	43.8	21.0	23.6	28.3	43.6	5.4	7.1
GM-SCAD	42.3	43.8	20.3	23.9	40.0	54.7	4.9	5.9
GM-MCP	42.5	43.8	20.3	23.4	14.4	33.6	6.4	9.9
GNSC	42.8	44.0	0.0	19.1	13.3	89.2	5.9	12.7
GADA	42.6	43.6	10.8	19.5	12.8	68.9	5.9	12.6
GSCAD	42.9	44.5	0.0	19.2	18.9	82.5	4.6	11.8
GMCP	42.7	43.9	16.5	21.8	17.8	52.8	6.1	12.6
GM-GNSC	42.6	43.8	22.9	26.4	45.0	64.4	4.8	5.8
GM-GADA	42.6	43.6	23.1	26.0	23.9	48.1	6.0	8.0
GM-GSCAD	42.8	43.8	24.4	26.7	45.6	59.2	5.0	5.8
GM-GMCP	42.2	43.4	23.4	25.9	13.3	26.9	8.1	11.8

**Table 8:** Three groups with class-imbalance (*π*_1_ = 0.4, *π*_2_ = 0.2, *π*_3_ = 0.4), dense block diagonal structure (*ρ* = 0.9) and small mean difference (*γ* = 0.1).

Method	PA		g-mean		SEN		PPV	
	Median	Upper	Median	Upper	Median	Upper	Median	Upper
NSC	40.5	41.8	0.0	27.5	5.6	25.8	6.7	17.9
ADA	40.7	41.9	0.0	28.3	5.0	30.0	6.3	17.2
SCAD	40.5	41.7	0.0	28.1	6.1	65.6	6.3	13.8
MCP	40.4	41.7	12.3	28.9	4.4	37.8	7.0	17.0
GM-NSC	38.7	40.1	31.2	33.0	61.7	86.9	4.4	5.2
GM-ADA	38.8	40.0	30.7	32.7	39.4	68.3	5.2	6.4
GM-SCAD	38.8	40.2	31.1	32.7	54.4	77.8	4.6	5.3
GM-MCP	39.3	40.3	29.5	31.9	17.8	50.0	6.2	9.1
GNSC	40.6	41.9	0.0	27.6	3.3	30.0	7.3	20.2
GADA	40.4	41.6	0.0	28.6	3.3	22.8	7.9	20.0
GSCAD	40.6	41.7	0.0	28.5	5.0	65.3	5.5	14.8
GMCP	40.1	41.2	21.4	30.0	5.0	26.1	7.2	17.3
GM-GNSC	38.6	39.5	30.8	32.4	55.6	81.4	4.6	5.8
GM-GADA	38.9	39.7	30.7	32.5	35.6	70.3	5.1	7.3
GM-GSCAD	38.6	39.5	31.3	32.8	48.3	75.6	4.7	5.5
GM-GMCP	38.9	39.9	30.5	32.2	25.0	53.6	6.0	9.6

## Real data study

In this section, we applied conventional NSC and the proposed methods (ALT-NSC and GEN-NSC) to four real microarray data sets. The main characteristics of the four microarray data sets are presented in Table 9.

**Table 9:** Characteristics of the real microarray data sets.

Author	Reference	Disease	Class	Gene	Sample
Gravier et al. (2010)	[20]	Breast cancer	2	2905	168
Pomeroy et al. (2002)	[21]	CNS cancer	4	5597	38
Yeoh et al. (2002)	[22]	Leukemia	6	12625	248
Ramaswamy et al. (2001)	[23]	Cancer	14	16063	198

CNS: central nervous system.

The Gravier et al. [20] data set came from a breast cancer study that consists of 111 patients with no events and 57 patients with early metastasis after diagnosis. The Pomeroy et al. [21] data set is a CNS cancer study that consists of 10 medulloblastomas, 10 CNS AT/RTs (renal and extrarenal rhabdoid tumors), 8 supratentorial PNETs and 10 non-embryonal brain tumors (malignant glioma). The Yeoh et al. [22] data set is a acute lymphoblastic leukemia (ALL) study that consists of six types of pediatric ALL subtypes: 43 T-cell lineage ALL (T-ALL), 27 E2A-PBX1, 79 TEL-AML1, 20 MLL rearrangements, 15 BCR-ABL, and 64 hyperdiploid karyotypes with more than 50 chromosomes (HK50). The Ramaswamy et al. [23] data set consists of 14 types of cancer samples as follows: 12 breast adenocarcinoma, 14 prostate adenocarcinoma, 12 lung adenocarcinoma, 12 colorectal adenocarcinoma, 22 lymphoma, 11 bladder transitional cell carcinoma, 10 melanoma, 10 uterine adenocarcinoma, 30 leukemia, 11 renal cell carcinoma, 11 pancreatic adenocarcinoma, 12 ovarian adenocarcinoma, 11 pleural mesothelioma and 20 central nervous system.

We randomly split each data set into a training set and a test set with 33% of the data allocated to the test set. This process was iterated 100 times. We chose optimal tuning parameters (*a*, *λ*) as the values that give the maximum of 5-fold CV prediction accuracy or g-mean under the training data set. We compared prediction accuracy, g-mean, AUC (only for the Gravier data set) and the number of selected genes.

The Gravier data set is slightly imbalanced; the proportions of “no events” and “early metastasis” are 0.66 and 0.34. There was no improvement of PA, g-mean and AUC by the proposed methods, but using the alternative penalty functions reduced N-sig. MCP had the smallest N-sig (Table 10). The Pomeroy data set is balanced. ALT-NSC improved g-mean, but not PA, and reduced N-sig, with the exception of SCAD. GM did not improve either PA or g-mean. MCP performed the best with higher PA and g-mean and smaller N-sig compared to NSC. GMCP performed very similarly to MCP with slightly inferior prediction performance but much smaller N-sig (Table 11). The Yeoh data set is imbalanced. Like the Golub data set [24], the prediction was easy for this data set despite the large number of classes. PA and g-mean were not improved, but N-sig was reduced by the proposed methods. Both ALT-NSC and GEN-NSC reduced N-sig, with GMCP having the smallest N-sig. GMCP selected 418 genes, while NSC selected 1456 genes at the median quartile (Table 12). The Ramaswamy data set has a large number of classes, and, as a result, all the classifeirs had low g-mean values. All the MCP methods and GM-SCAD had positive g-mean values, but the other methods had zero as the 90% quantile of g-mean (Table 13).

**Table 10:** Gravier (2010) data set: Breast cancer study with 2 classes.

Method	PA		g-mean		AUC		N-sig	
	Median	Upper	Median	Upper	Median	Upper	Median	Upper
NSC	75.0	78.6	68.5	73.9	79.5	83.5	685	1176
ADA	75.0	78.6	68.5	73.9	79.9	83.3	550	1174
SCAD	75.0	78.6	69.3	73.9	79.9	83.3	928	1550
MCP	73.2	78.6	67.4	73.0	78.5	82.5	370	863
GM-NSC	75.0	78.6	70.5	75.7	80.9	83.8	660	968
GM-ADA	75.0	78.6	68.8	75.2	80.3	83.8	475	859
GM-SCAD	75.0	78.6	70.0	74.6	80.8	83.7	692	1196
GM-MCP	73.2	78.6	67.5	73.9	79.8	83.5	458	858
GNSC	75.0	78.6	70.4	74.1	80.0	83.5	720	1794
GADA	75.0	78.6	68.5	73.9	79.7	83.4	548	1198
GSCAD	75.0	78.6	68.5	73.9	80.0	83.2	908	1570
GMCP	73.2	78.6	67.5	72.2	78.4	82.9	435	1071
GM-GNSC	75.0	78.6	70.4	74.5	80.8	83.9	681	1396
GM-GADA	75.0	78.6	69.1	73.9	80.5	83.5	516	1007
GM-GSCAD	75.0	78.6	69.6	74.6	81.1	84.1	733	1191
GM-GMCP	75.0	78.6	68.5	73.5	79.7	83.3	480	848

“PA”, “g-mean” and “AUC” are accuracy, geometric mean and AUC of class prediction, calculated from the test data set. “N-sig” is the number of selected genes from the training data set. “Median” and “Upper” are median and upper quartiles of 100 repetitions. The scale of all the numbers is a percentage.

**Table 11:** Pomeroy (2002) data set: CNS study with 4 classes.

Method	PA		g-mean		N-sig	
	Median	Upper	Median	Upper	Median	Upper
NSC	80.2	84.6	68.7	76.0	422	2706
ADA	81.8	84.6	76.0	81.6	383	1911
SCAD	83.3	85.7	76.0	84.1	822	2776
MCP	83.3	91.7	76.0	85.7	212	584
GM-NSC	81.8	84.6	70.7	81.6	1264	4679
GM-ADA	83.3	84.6	76.0	81.6	480	2905
GM-SCAD	83.3	85.7	76.0	84.1	1183	3206
GM-MCP	83.3	91.7	76.0	84.1	212	584
GNSC	78.6	83.7	67.2	76.0	500	2378
GADA	81.8	84.6	68.7	81.6	378	1683
GSCAD	81.8	84.6	69.7	81.6	714	2574
GMCP	83.3	90.9	76.0	84.1	52	228
GM-GNSC	81.8	84.6	69.7	81.6	1442	3929
GM-GADA	81.8	84.6	70.7	82.3	448	2063
GM-GSCAD	81.8	84.6	70.7	82.3	1184	3018
GM-GMCP	83.3	85.7	76.0	84.1	54	267

**Table 12:** Yeoh (2002) data set: Leukemia study with 6 classes.

Method	PA		g-mean		N-sig	
	Median	Upper	Median	Upper	Median	Upper
NSC	95.2	96.7	91.3	95.6	1456	2050
ADA	95.2	97.5	91.9	95.6	1044	2152
SCAD	95.2	96.4	92.1	94.8	1451	1991
MCP	95.2	96.4	93.9	95.5	1022	1454
GM-NSC	95.2	96.4	91.8	95.0	2168	3847
GM-ADA	95.2	97.5	94.0	95.6	2112	2310
GM-SCAD	95.2	96.4	92.2	94.8	1834	4072
GM-MCP	95.1	96.4	92.1	94.8	1454	2449
GNSC	96.3	96.4	93.9	95.6	690	1114
GADA	95.2	97.6	93.9	95.6	642	1041
GSCAD	96.3	97.6	94.0	95.6	990	1312
GMCP	96.4	97.6	93.5	95.6	418	496
GM-GNSC	95.2	96.4	93.9	95.6	931	1528
GM-GADA	95.2	96.7	94.0	95.6	820	1290
GM-GSCAD	96.3	96.4	93.5	95.3	1154	1998
GM-GMCP	96.4	97.6	94.4	95.6	484	911

**Table 13:** Ramaswamy (2001) data set: Cancer study with 14 classes.

Method	PA		g-mean		N-sig	
	Median	Upper	Median	90%*	Median	Upper
NSC	70.0	75.0	0.0	0.0	1570	4430
ADA	71.9	76.9	0.0	0.0	1346	3069
SCAD	70.6	75.4	0.0	0.0	2414	5233
MCP	72.3	77.4	0.0	62.1	1157	2566
GM-NSC	63.9	72.3	0.0	0.0	4610	16063
GM-ADA	69.7	76.9	0.0	0.0	2313	10264
GM-SCAD	68.7	75.8	0.0	55.6	6174	14779
GM-MCP	72.1	78.5	0.0	62.7	1575	4535
GNSC	68.7	73.8	0.0	0.0	396	1205
GADA	69.2	75.4	0.0	0.0	306	1042
GSCAD	68.2	73.6	0.0	0.0	539	5160
GMCP	70.3	77.0	0.0	0.0	206	578
GM-GNSC	18.5	68.7	0.0	0.0	6	16063
GM-GADA	60.3	71.6	0.0	0.0	466	16037
GM-GSCAD	60.9	70.4	0.0	0.0	3027	15757
GM-GMCP	69.2	75.5	0.0	58.3	234	3853

90%*: ninty percent quantile

## Discussion

In this article, we proposed several variations of NSC that use alternative genewise shrinkages. We derived these methods using three penalized regression models that enjoy oracle properties and have closed-form solutions under an orthonormal design. We also further modified these variants of NSC by adapting genewise penalty functions that use the correlations between the parameters belonging to the same gene, and the geometric mean approach for class-imbalanced data. We showed that these methods have better performance than conventional NSC in terms of prediction accuracy, g-mean and gene selection through simuation and real data studies.

We conducted simulation studies to evaluate the proposed methods. We used a block diagonal covariacne matrix with the block being an auto-regresive structure with a paramter *ρ*. When *ρ* is small, the block matrix becomes sparse, and thus it behaves like an identity matrix. Ohterwise, when *ρ* is large, the block matrix becomes dense, and thus it behaves like a block exchageable matrix. We varied *ρ*, the degree of class imbalance and the effect size of DE genes. The proposed methods had better peformance in terms of prediction accuracy and gene selection compared to NSC when the block matrix was dense and class was imbalanced. When the effect size was moderate, ALT-NSC methods performed well and among those MCP performed the best. When the effect size is small, GM method performed well with the highest g-mean.

We applied the proposed methods to four real microarray data sets. The proposed methods improved the g-mean, but not the overall prediction accuracy, in the data sets we considered. When the number of classes was two (Gravier data set) or prediction was easy (Yeoh data set), only gene selection was improved by the alternative penalty functions. In the data set with the moderate number of classes (Pomeroy data set), g-mean was improved by the alternative penalty functions. When the data set had very large number of classes (Ramaswamy data set), using the genewise penalty functions reduced the performance.

In many applications, it is desirable to develop classifiers that use the smallest possible number of genes. For example, one may wish to use an RT-PCR assay to discriminate between different types of tumors or to determine the prognosis of a patient with a given tumor type. Such an assay will be prohibitively expensive if the expression levels of more than a handful of genes are needed. Thus, a classification method that produces comparable accuracy to another method using fewer genes would be considered superior in these situations. Hence, the fact that our proposed methods consistently use fewer genes than conventional NSC represents a significant advantage of our methods even if prediction accuracy is not always improved. MCP would be very useful in real applications becacuse they have shown to select the most reliable parsimonious gene set with competitive predictive accuracy.

Both simulation and real data studies showed that our proposed methods produced greater improvement compared to conventional NSC in the data sets with three or four classes, but not in data sets with very large numbers of classes. When the number of classes is large, the sample size per class is usually small, and this affects the efficiency of shrunken mean estimators. By the virtue of the oracle property, ALT-NSC can produce more efficient estaimtes of the shrunken means, which yields better performance on both prediction and gene selection. Genewise shrinkages also improve the NSC classifier by combining the related genes in the same class, producing more accurate estimates when the size of the class is small (which commonly occurs when the number of classes is large). Clearly, the genewise penalty (GEN-NSC) shrinks a mean estimator toward zero faster than the non-genewise penalty (ALT-NSC), as shown in the simulations and the real data study. Appropriately fast shrinkage will be able to remove noisy genes effectively. However, one observes that when the number of classes is large, such as the Ramaswamy data set, the amount of shrinkage produced by the genewise penalty is so large that NSC loses some prediction accuracy. Thus, applying GEN-NSC to data sets with too many classes may not be recommended when the objective is to maximize predictive accuracy (rather than minimize the number of selected genes).

The performance of the methods can be affected by heterogeneity of gene expression, and this heterogeneity happens when the variances of genes differ by groups. This was pointed out by Tibshirani et al. [2] and was observed in the compariative study of Lee et al. [25]. Tibshirani et al. [2] suggested an ad-hoc method to account for heterogeneity. However, that method is only applicable in the case where class centroids are not separated. The method of Pang et al. [6] may overcome this problem because it combines the linear and quadratic discriminant scores, where the latter assumes unequal variances by classes. Since the method does not perform varaible selection, applying the mean shrinkage to their method would be a future research to handle this heterogeneit problem.

## Supporting information

S1 Rscript R source code. This file contains the R functions that implement ALT-NSC and GEN-NSC.
(ZIP)
